# The compensatory enrichment of sphingosine -1- phosphate harbored on glycated high-density lipoprotein restores endothelial protective function in type 2 diabetes mellitus

**DOI:** 10.1186/1475-2840-13-82

**Published:** 2014-04-21

**Authors:** Xunliang Tong, Pu Lv, Anna V Mathew, Donghui Liu, Chenguang Niu, Yan Wang, Liang Ji, Jizhao Li, Zhiwei Fu, Bing Pan, Subramaniam Pennathur, Lemin Zheng, Yining Huang

**Affiliations:** 1 Department of Neurology, Peking University First Hospital, Beijing 100034, China; 2 The Institute of Cardiovascular Sciences and Institute of Systems Biomedicine, School of Basic Medical Sciences, and Key Laboratory of Molecular Cardiovascular Sciences, Ministry of Education, Key Laboratory of Cardiovascular Molecular Biology and Regulatory Peptides, Ministry of Health, Peking University Health Science Center, Beijing 100191, China; 3 Department of Internal Medicine, University of Michigan, Ann Arbor, Michigan 48109, USA; 4 Division of cardiology, Zhongshan Hospital affiliated to Xiamen University, Xiamen heart center, Xiamen, Fujian Province 361004, China

**Keywords:** Sphingosine 1 phosphate, High density lipoprotein, Type 2 diabetes mellitus, Cyclooxygenase-2, Endothelial cells

## Abstract

**Background:**

Glycation of high-density lipoprotein (HDL) decreases its ability to induce cyclooxygenase-2 (COX-2) expression and prostacyclin I-2 (PGI-2) release in endothelial cells. Whether lipid content of HDL, especially sphingosine-1-phosphate (S1P), plays any specific role in restoring the protective function of HDL in type 2 diabetes mellitus (T2DM) is still unknown.

**Methods and results:**

Immunochemical techniques demonstrated that glycated HDL loses its protective function of regulating COX-2 expression compared with diabetic HDL. We proved that the lipid content, especially phospholipid content differed between diabetic HDL and glycated HDL. Levels of HDL-c-bound S1P were increased in T2DM compared with control subjects as detected by UPLC-MS/MS (HDL-c-bound S1P in control subjects vs. T2DM: 309.1 ± 13.71 pmol/mg vs. 382.1 ± 24.45 pmol/mg, P < 0.05). Additionally, mRNA levels of S1P lyase enzymes and S1P phosphatase 1/2 were decreased in peripheral blood by real-time PCR. Antagonist of S1P receptor 1 and 3 (S1PR1/3) diminished the functional difference between apoHDL&PL (HDL containing the protein components and phospholipids) and diabetic apoHDL&PL (diabetic HDL containing the protein components and phospholipids). With different doses of S1P reconstituted on glycated HDL, its function in inducing the COX-2 expression was restored to the same level as diabetic HDL. The mechanism of S1P reconstituted HDL (rHDL) in the process of regulating COX-2 expression involved the phosphorylation of ERK/MAPK-CREB signal pathway.

**Conclusion/Significance:**

S1P harbored on HDL is the main factor which restores its protective function in endothelial cells in T2DM. S1P and its receptors are potential therapeutic targets in ameliorating the vascular dysfunction in T2DM.

## Introduction

Diabetes mellitus is a systemic metabolic disease and a major risk factor for the development of atherosclerosis [1,2]. Hyperglycemia causes alterations in lipid metabolism which bring about a series of adverse effects, including enhanced HDL clearance, decreased apoA-1 transcription and accelerated HDL glycation [3,4]. These dramatically altered lipid metabolic changes can promote atherosclerosis [5,6].

Native HDL has protective role in inducing cyclooxygenase-2 (COX-2) expression and prostacyclin I-2 (PGI-2) release in endothelial cells [7]. PGI-2 is the major COX catalyzed product which is metabolized from arachidonic acid (AA) and plays a protective role in the macrovascular endothelium [7,8]. Previous studies have shown that the lipid component of HDL can induce COX-2 expression and PGI-2 release, especially sphingosine-1-phosphate (S1P) [9]. S1P is primarily carried on HDL in plasma and has many biological functions, such as ability to promote vasodilation, vasoconstriction, and angiogenesis, protect against ischemia/reperfusion injury, and inhibit/reverse atherosclerosis [10]. The level of S1P is controlled by two pathways, dephosphorylation of S1P regenerating sphingosine by S1P-specific phosphatases (SGPP), and the other is the irreversible cleavage by sphingosine-1-phosphate lyase (SPL) [11-14].

Non-enzymatic glycation is a common modification that occurs in type 2 diabetes mellitus (T2DM), which could impair protein function [15,16]. Previous studies showed that non-enzymatic glycation of HDL impairs its anti-inflammatory function and vascular protective effects [17-19]. Diabetic HDL could also down-regulate expression of SR-BI causing a dysfunction in proliferation and migration of endothelial cells [20]. Previous studies have shown alteration of sphingosine metabolism in diabetes mellitus [21-23], so in this study, we focused on the effect of the lipid component of HDL after glycation impairment. We recently proved that HDL-associated S1P was increased in T2DM than healthy volunteers [24]. In this work, we attempt to elucidate the mechanism of S1P’s protective function in inducing COX-2 expression and PGI-2 release.

## Methods

### Ethical approval

Healthy control subjects and patients with type 2 diabetes were recruited following informed consent. Human umbilical vein endothelial cells (HUVECs) were obtained by collagenase digestion of umbilical cords which were donated by the volunteers after written informed consent. The study was approved and supervised by the Institutional Review Board and Ethics Committee of Peking University First Hospital (Beijing, China).

### Chemical agents

S1P, C17-D-erythro-sphingosine-1-phosphate (C17-sph) and the antagonist of S1PR1 and S1PR3 were purchased from Avanti polar lipids (Alabama, USA). The antibodies for western blot against phospho-extracellular regulated kinase (ERK) 1/2 and cAMP-response element binding protein (CREB) were purchased from Cell Signal Technology, Danvers, MA. The antibodies of phospho-p38 mitogen-activated protein kinase (MAPK), p38 MAPK and β-actin were purchased from Santa Cruz Biotechnology, Santa Cruz, CA. Antibody against COX-2 and competitive enzyme immunoassay kit for 6-keto PGF1α were from Cayman Chemical (Michigan, IL). Horseradish peroxidase (HRP)-goat-anti-rabbit IgG and HRP-goat-anti-mouse IgG were purchased from MBL (Nagoya, Japan). Endothelial cell medium (ECM) was purchased from ScienCell Research Laboratories (Carlsbad, CA).

### Subjects

Healthy volunteers and patients underwent physical examination, laboratory tests and ultra-sound examination of the large arteries. The patients were all first diagnosed with T2DM in accordance with international standards; fasting plasma glucose (FPG) ≥ 7.0 mmol/L and glycated hemoglobin (HbA1c) greater than 6.5%. The patients were all first diagnosed in the hospital before initiation of treatment. The healthy subjects had no family history of diabetes, and they had normal FPG level and normal glucose tolerance. Allowing the criteria above, 15 T2DM and 15 healthy patients were recruited. The detailed information of the recruited patients and healthy volunteers has been described in a previous study [24].

### Cell culture

Human umbilical vein endothelial cells (HUVECs) were obtained by collagenase treatment of umbilical cord vein as described previously [25]. Cells were cultured on gelatin-coated dishes and propagated in endothelial cell medium supplemented with 5% FBS and endothelial cells were grown at 37°C in an incubator with humidified air containing 5% CO_2_. HUVECs were harvested when cells reached 70–80% confluent and passages three to fifty were used.

### Blood collection and HDL isolation

The fresh blood from T2DM and healthy volunteers was drawn after overnight fasting into EDTA-Na_2_ vacuum tubes. Plasma was separated by centrifuging at 2,500 rpm at 4°C for 15 minutes. HDL (1.063-1.210 g/ml) was isolated by ultracentrifugation as described previously [26]. HDL2 (1.063 < d < 1.125 g/ml) and HDL3 (1.125 < d < 1.21 g/ml) were isolated by sequential ultracentrifugation as previously described [27]. HDL sub fractions were dialyzed against saline/EDTA (150 mM NaCl, 300 μM EDTA, pH 7.4), sterilized by filtering through a 0.22 μm membrane, and stored at 4°C until needed. The HDL was used within a month after isolation. The purity of the HDL was confirmed by SDS-PAGE and western blot using goat anti-apoA-I polyclonal antibody (DiaSorin, Stillwater, OK) and quantified through the measurement of apoA-I content by nephelometry (Dimension XPand, Dade Behring, Germany). Equal concentration of apoA-1 from isolated HDL was used for cell treatments and S1P level determination.

### Modification, selected delipidation and reconstitution of HDL

HDL was incubated in glucose/phosphate-buffer saline (PBS) buffer with butylated hydroxytoluene (BHT) (the final concentration of glucose is 25 mmol/L) at 37°C for 7 days in vitro [17]. During this procedure, the oxidation levels of modified HDL were measured (Additional file 1: Figure S1). After HDL was dialyzed with PBS, glycated HDL was used for cell experiments and reconstitution. Selective delipidation was achieved as described previously. HDL was agitated with di isopropyl ether in a ratio of 1:2 (vol/vol) at 4°C for 24 h [28]. After extraction, the mixture was centrifuged at 2,000 rpm for 5 minutes to separate the aqueous and organic phase. Apo HDL&PL to (HDL containing the protein components and the phospholipids) was collected in the aqueous phase and PL-depleted HDL-lipids (the lipid component of HDL except for the phospholipids) in the organic phase. Alternatively, HDL was agitated with a mixture of butanol and di isopropyl ether (vol/vol, 40:60) in a ratio of 1:2 (vol/vol) for 30 minutes at the room temperature. After centrifugation we collected apoHDL (HDL containing the protein components only) in aqueous phase and HDL-lipids (all lipid component of HDL) in organic phase. ApoHDL&PL and apoHDL were filtered before use. Reconstituted HDL (rHDL) was made by adding S1P to glycated HDL at the desired concentration and then mixed by rotation overnight at 4°C for further usage [29]. The levels of S1P used for reconstituting were nearly equal to the S1P we detected on reconstituted HDL (Additonal file 1: Figure S2).

### S1P extraction and detection by UPLC-MS/MS

UPLC-MS/MS technique was employed to measure the levels of HDL-associated S1P as previously described. Serum samples mixed with internal standard C17-sph (50 μl of 1000 μg/L) was precipitated by methanol at the volume ratio of 1:4. After centrifugation at 12,000 rpm for 15 minutes, the supernatant was collected for UPLC-MS/MS analysis performed by a Waters ACQUITY UPLC^TM^ system as described previously [24]. Waters ACUITY UPLC BEH Phenyl column (1.8 μm; 2.1 mm × 100 mm) was selected for chromatographic separation. The injection volume was 5 μL. Methanol (A) and 0.5% formic acid in ultrapure water (B) were used as mobile phases. The gradient started at 10% A and then increased linearly to 60% in 6 minutes, and then to 100% at 6 minutes and kept for 2 minutes, followed by a decrease to initial conditions of 10% A and held for 2 minutes to allow for equilibration. The flow rate was 0.3 mL/min. The column was maintained at 40°C, and the sample room temperature was 10°C.

### Real-time PCR assay for SPL and SGPP1/2

Real-time PCR was performed to determine mRNA levels of SPL and SGPP1/2 from peripheral blood of the patients and healthy volunteers. Total RNA was extracted using the TRIzol reagent (Invitrogen, USA) and reverse transcription was performed using an RT-PCR kit (TransGen Biotech, China). Real-time experiments were conducted on a DNA Engine Opticon System (MJ research Inc, USA) using SYBR Green PCR Master Mix kit in triplicate specific primers. The sequences of primers to determine the expression of the target gene were as follows: SPL [5’-CTTGATGCACTTCGGTGAGA-3’ (forward); 5’-TCCACCCCTTAGCAGTCATC-3’ (reverse)], SGPP1 [5’-ACCGCCATCCCCATTTCT-3’ (forward); 5’-AGGAATCCAGCAATAATATCCAG-3’ (reverse)], SGPP2 [5’-gTATTATACTCATGGTTCAAGGTG-3’ (forward); 5’-GTGTAGGTAACAAACTTGTAAGG-3’ (reverse)] and Glyceraldehyde 3-phosphate dehydrogenase (GAPDH) [5’-CGGAGTCAACGGATTTGGTCGTAT-3’ (forward); 5’-AGCCTTCTCCATGGTGGTGAAGAC-3’ (reverse)]. The PCRs consisted of 5 min at 95°C followed by 40 cycles of denaturation for 30 sec at 95°C, annealing for 30 sec at 56°C and a primer extension for 30 sec at 72°C. The comparative CT method was used to quantify the expression of SPL, SGPP1 and SGPP2 using GAPDH as the normalized control.

### ELISA: quantitation of 6-keto PGF1α

PGI-2 is non-enzymatically hydrated to 6-keto PGF1α and estimation of systemic PGI-2 production has often been assessed by measurement of 6-keto PGF1α [30]. The cell culture supernatants were collected after centrifugation at 3000 rpm for 15 minutes. For accurate measurement of PGI-2 production, 6-keto PGF1α in cell culture supernatants were determined using enzyme immunoassay kit (Cayman Chemical). The final results were normalized to cell protein concentration.

### Western blotting and EMSA

Western blot analysis was performed as described previously [31], cells were washed twice with cold 1X PBS and lysed on ice for 30 min in lysis buffer. The lysates were subjected to centrifugation at 12,000 rpm for 15 min at 4°C and the supernatant was utilized for analysis. Protein concentrations were determined using BCA method. The boiled samples were loaded on Ready SDS-10% PAGE gels and used for Western blot analysis with the protein- specific antibody. HRP- labeled secondary anti-body was used for detection of signal by electrochemiluminescence from Pierce (California, USA).

DNA-protein binding reactions were performed by incubating nuclear extracts with specific CREB-CIE (cis-inducible element) DNA binding. Polyclonal anti-CREB antibody was added to the reaction mixture containing the labeled probe. EMSA was performed as previously described [32].

For EMSA assay, 10 μg nuclear protein was used with 15 fmol of HRP-end-labeled double stranded oligonucleotides in mixed nuclear extraction from HUVEC after treated with HDL as described above. The sequence of oligonucleotide containing CREB was: 5’-AGA GAT TGC CTG ACG TCA GAC AGC TAG-3. Oligonucleotides were end-labeled with HRP polynucleotide kinase and purified on G-50 columns (Roche Diagnostics). The DNA binding reaction of HRP labeled double-stranded oligonucleotides was performed at room temperature for 20 min, according to the manufacturer's protocol. Electrophoretic mobility shift assays were performed with the Light Shift Chemiluminescent Kit from Pierce, according to the manufacturer's recommendations.

### Statistical analyses

All experiments were repeated in triplicate if not mentioned. Data are presented as mean ± SEM unless indicated otherwise. Differences were compared with two-tailed Student’s *t*-test or one-way ANOVA using GraphPad Prism software. p < 0.05 was considered statistically significant.

## Results

### Lipid component of diabetic HDL improved the up-regulation of COX-2 expression and PGI-2 release in endothelial cells

**Figure 1:**
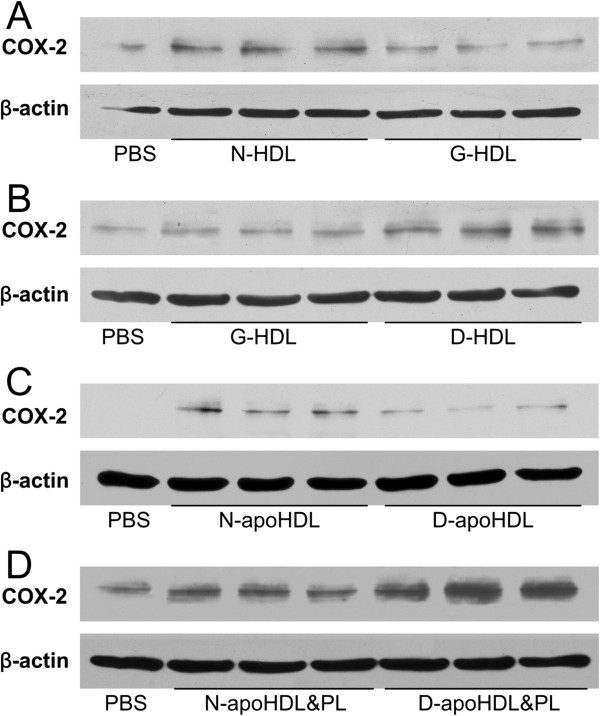
**The phospholipids component of HDL from T2DM enhanced COX-2 expression and PGI-2 release. A-B**: glycated HDL (equal to 30 μg/ml of HDL) loss its function in regulation COX-2 expression compared with N-HDL **(A)** and diabetic HDL (equal to 30 μg/ml of HDL) has more increased COX-2 expression than glycated HDL (equal to 30 μg/ml of HDL) **(B)**. C-D: HDL only containing protein components without any the lipid from N-HDL (N-apoHDL) (equal to 30 μg/ml of HDL) is more functional than it’s from diabetes mellitus (D-apoHDL) (equal to 30 μg/ml of HDL) in regulating COX-2 expression **(C)** and the preservation of phospholipids and protein components of HDL from type 2 diabetes mellitus, D-apoHDL&PL (equal to 30 μg/ml of HDL), reversed the function in regulating COX-2 expression compared with the control subjects, N-apoHDL&PL (equal to 30 μg/ml of HDL) **(D)**. Each experiment was repeated three times.

To test whether the glycation modification impaired HDL function in endothelial cells, we used native HDL and non-enzymatically glycated HDL treated with HUVECs at the final concentration of 30 μg/ml for 6 hours. Glycated HDL failed to induce COX-2 expression (Figure 1A). Compared with glycated HDL, the diabetic HDL showed significantly increased induction of COX-2 expression (Figure 1B). To eliminate the differences in lipid component, delipidated HDL was used to treat HUVECs and the delipidated apoHDL regulated COX-2 expression slightly more than delipidated diabetic apoHDL. Both apoHDL and diabetic apoHDL partially lost the ability in up-regulating COX-2 expression (Figure 1C), which suggests that such effects are in part mediated by lipid component instead of protein. The selectively delipidated HDL, apoHDL&PL were used to incubate with HUVECs at the final protein concentration of 30 μg/ml for 6 hours. Diabetic apoHDL &PL restored the effects of inducing COX 2 expression compared with apoHDL&PL (Figure 1D), which implies that the phospholipid (PL) component was essential for these effects.

### S1P levels are increased in lipid component of HDL in diabetics compared to controls

**Figure 2:**
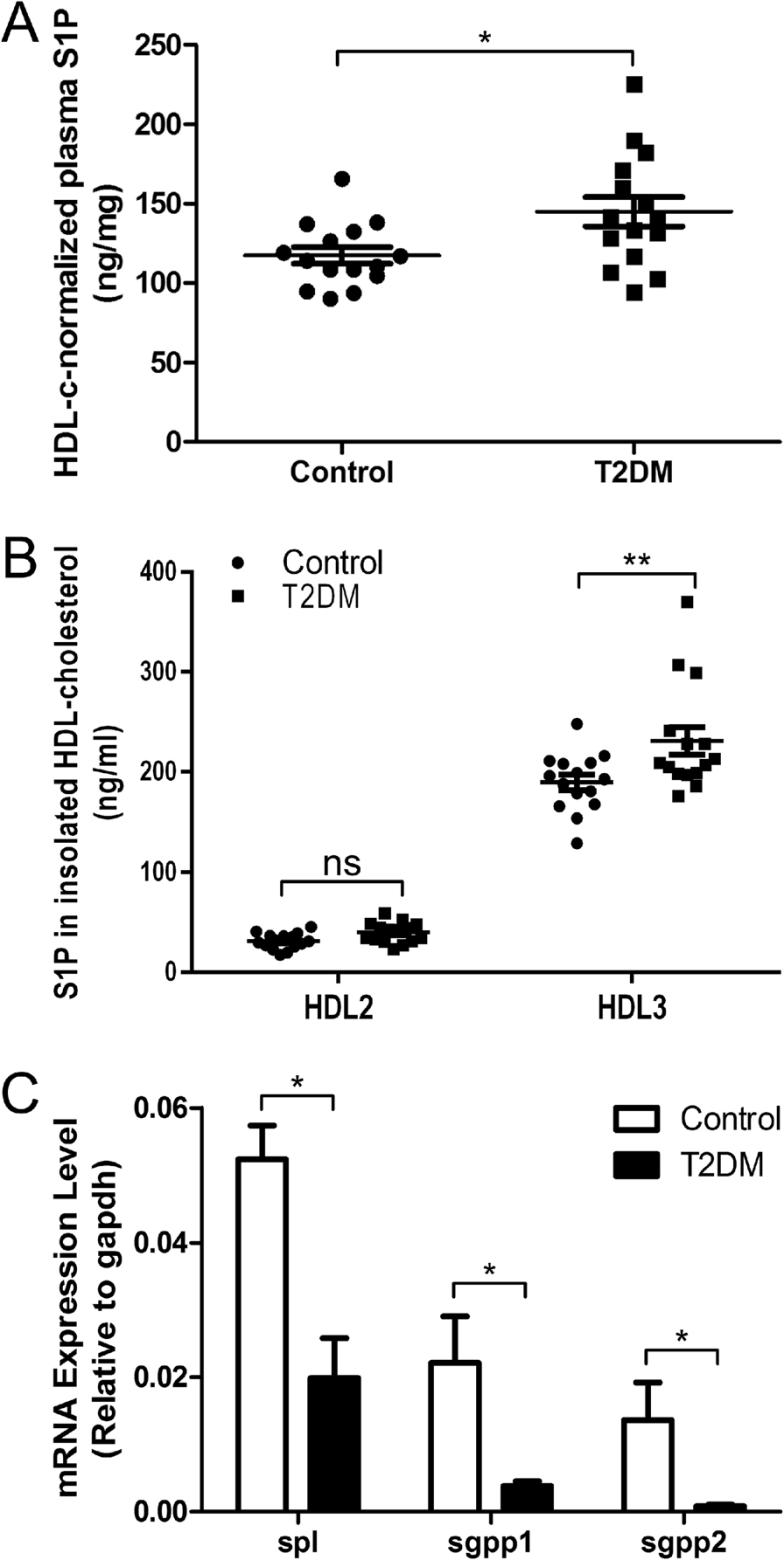
**Levels of S1P and its related enzymes were detected by UPLC-MS/MS and real-time PCR.** Levels of HDL-bound S1P were significantly increased in diabetes mellitus than control subjects. **A-B**: S1P level was normalized by HDL-c in plasma between control subjects and T2DM **(A)**, levels of diabetic HDL3- associated S1P were increased compared with native HDL3-associated S1P **(B)**. *P < 0.05 vs. healthy group. The levels of mRNA involved in S1P metabolism was measured by real-time PCR. **C**: The mRNA level of SPL and SGPP1/2 was detected in T2DM and control subjects. Mann–Whitney *U* test. Bars show medians. *p < 0.05.

To investigate the S1P level in plasma and HDL, we examined samples from 15 T2DM patients and control subjects using UPLC-MS/MS. As S1P in plasma was mainly bound to HDL-C, we normalized the levels of total plasma S1P to plasma HDL-C, which gave us the level of HDL-bound S1P. There was an increased HDL-C-normalized level of S1P in diabetes mellitus compared with control subjects (Figure 2A, control subjects vs. T2DM: 309.1 ± 13.71 pmol/mg vs. 382.1 ± 24.45 pmol/mg, P < 0.05). The levels of S1P in HDL2 and HDL3 were determined. The results showed that the levels of S1P associated with diabetic HDL3 were significantly higher than native HDL3, and S1P in diabetic HDL2 and native HDL2 showed no difference (Figure 2B, T2DM-HDL3 vs. control-HDL3: 189.7 ± 7.4 vs 230.9 ± 13.8 ng/mg, p < 0.01). The levels of S1P harbored on diabetic HDL (Y axis) were decreased with the increasing levels of HbA1c (Additional file 1: Figure S3). To investigate the mechanism for increased level of S1P from diabetic HDL, the expression of related enzymes that degenerate S1P was examined. The mRNA levels of SPL and SGPP 1/2 from peripheral blood was detected by the method of real-time PCR. The level of SPL was decreased 1.6 fold and the levels of SGPP1 and SGPP2 were also decreased 4.8 fold and 15.7 fold separately in diabetes compared with control subjects (Figure 2C).

### S1P receptor 1 (S1PR1) and S1P receptor 3 (S1PR3) antagonists diminished the effect of HDL derived lipids

**Figure 3:**
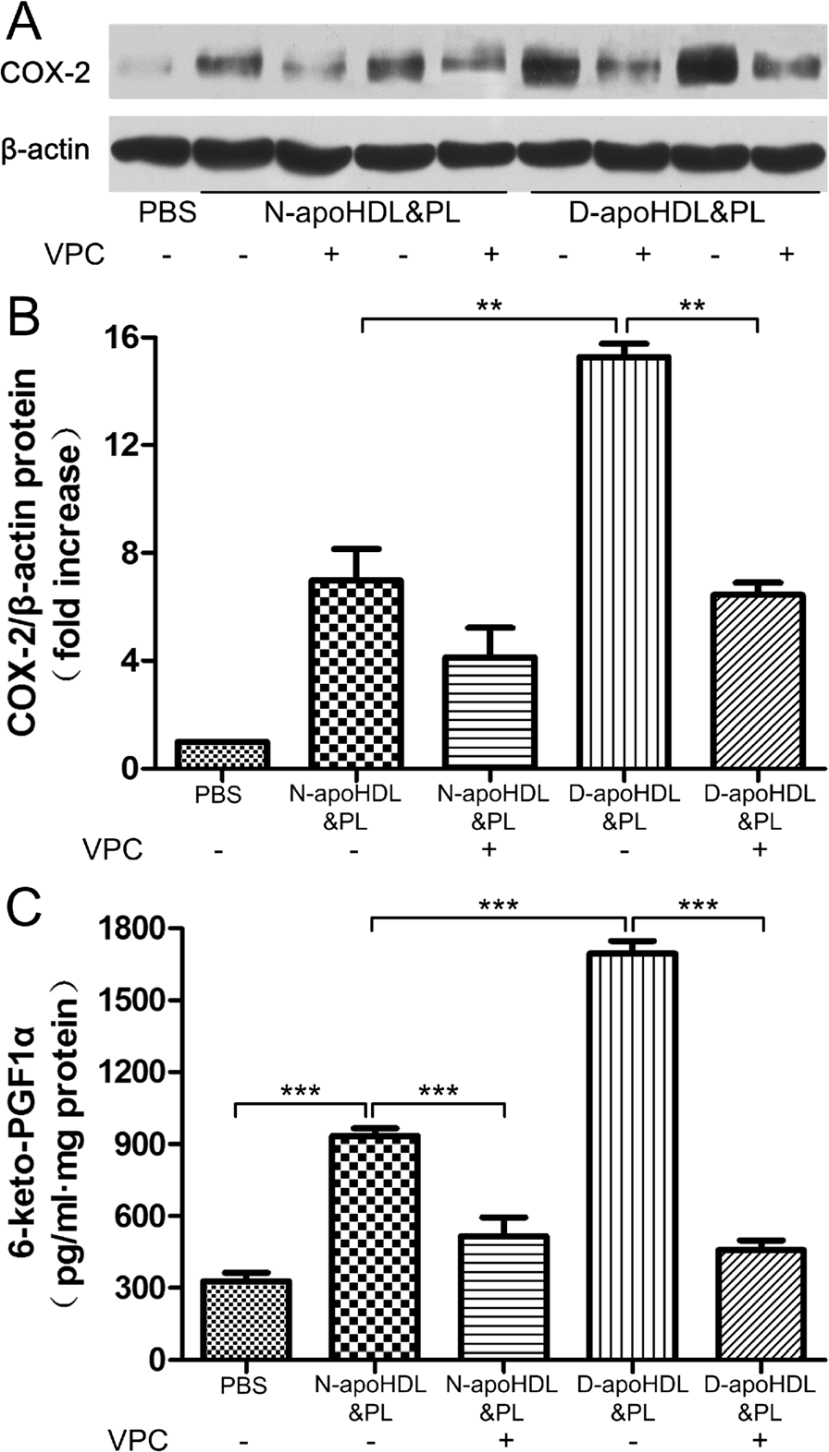
**The up-regulation of COX-2 expression by diabetic apoHDL&PL was blocked by VPC23019.** The up-regulation of COX-2 expression by diabetic apoHDL&PL was blocked by S1PR1 and S1PR3 antagonist (VPC23019). **A-B**: HUVECs were pre-incubated with or without VPC 23019 (15 nmol/ml) for 20 minutes and further separately stimulated with 30 μg/ml of N-apoHDL&PL or D-apoHDL&PL, expression of COX-2 was assayed by Western blotting **(A)**, the relative protein expression was normalized by β-actin **(B)** and the production of PGI-2 was determined by competitive ELISA **(C)**. Data are expressed as the means ± SEM of three independent experiments. Student’s *t* test. *p < 0.05. ***p < 0.001.

S1PR1 and S1PR3, specific receptors of S1P, are mainly located in the endothelial cells [14]. To investigate whether these two receptors were involved in this process, the S1PR1 and S1PR3 antagonist, VPC23019 was pre-incubated with the endothelial cells for 20 minutes at the final concentration of 2 nmol/L. The inhibitor diminished the effect of diabetic apoHDL&PL and apoHDL&PL in up-regulating COX-2 expression and PGI-2 release. (Figure 3A, B and C, apoHDL&PL vs. PBS, 933.4 ± 55.05 vs. 327.6 ± 61.80, pg · ml-1 · mg cellular protein-1, (P < 0.001) diabetic-apoHDL&PL vs. HDL&PL, 1695 ± 92.95 vs. 933.4 ± 55.05, pg · ml-1 · mg cellular protein-1, (P < 0.001); apoHDL&PL + VPC vs. apoHDL&PL, 517.1 ± 134.4 vs. 933.4 ± 55.05, pg · ml-1 · mg cellular protein-1, (P < 0.001); diabetic-apoHDL&PL + VPC vs. diabetic-apoHDL&PL, 457.9 ± 70.28 vs. 1695 ± 92.95, pg · ml-1 · mg cellular protein-1, (P < 0.001)). Therefore, the effects of S1P from diabetic HDL were likely mediated through the S1P receptors, S1PR1 and S1PR3.

### Reconstitution of S1P on glycated HDL was as effective as diabetic HDL in inducing COX-2 expression and PGI-2 release

**Figure 4:**
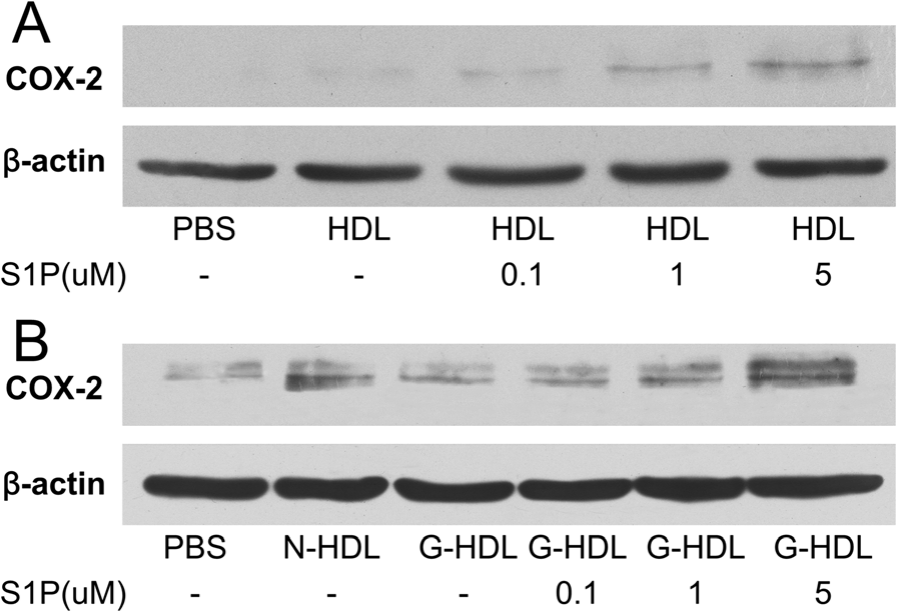
**S1P reconstituted on HDL repair the function of glycated HDL in a dose dependent manner. A-B**: S1P binding on HDL can enhance the COX-2 expression **(A)** and S1P restores the glycated HDL loss of function in inducing COX-2 expression **(B)** at 0.1 μM, 1 μM and 5 μM concentration of S1P (binding with 30 μg/ml HDL or glycated HDL). Each experiment was repeated three times.

We used different doses of free S1P for binding on HDL particles incubated with HUVECs and expression of COX-2 was induced by the reconstituted HDL in a dose-dependent manner (Figure 4A). The reconstitution of S1P on glycated HDL restored the ability of inducing COX-2 expression and PGI-2 release and was dose dependent (Figure 4B). These results show that S1P reconstituted on HDL can reverse the loss of function caused by glycation modification.

### Reconstituted HDL with addition of S1P on glycated HDL activates the ERK/MAPK pathways

**Figure 5:**
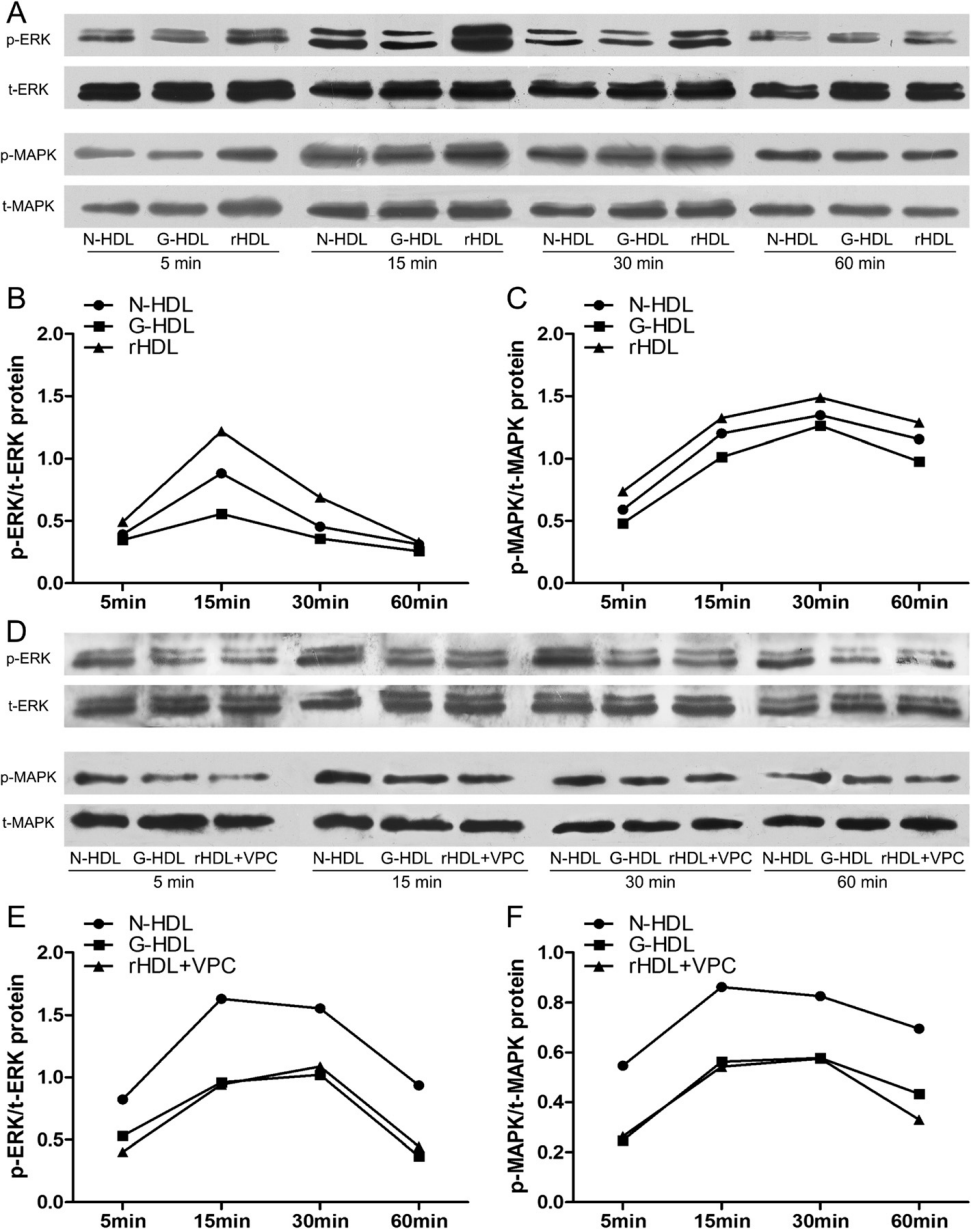
**Phosphorylation of ERK 1/2 and MAPK was induced by N-HDL, G-HDL, rHDL (G-HDL containing S1P). A-C**: HUVECs were incubated with 30 μg/ml of N-HDL, G-HDL, rHDL at each time point, 5 minute, 15 minute, 30 minute, and 60 minute. The activation of ERK1/2, p38 MAPK was analyzed by Western blotting (black circles = N-HDL, black squares = G-HDL, black bars = rHDL). **D-F**: HUVECs were pre-incubated with VPC 23019 (15 nmol/ml) for 20 minute and then treated with 30 μg/ml of N-HDL, G-HDL, rHDL at each time point, 5 minute, 15 minute, 30 minute, and 60 minute (black circles = N-HDL, black squares = G-HDL, black bars = rHDL + VPC). The activation of ERK1/2, p38 MAPK was analyzed by Western blotting. Representative bands from three independent experiments were shown.

The signal transduction activated by COX-2 expression involves the ERK/MAPK-CREB pathway (29). Three kinds of HDL, native HDL, glycated HDL (G-HDL) and the reconstituted HDL (rHDL, S1P added on glycated HDL) were used to treat HUVECs respectively. The glycated HDL was utilized to generate rHDL to reflect the increased glycation to HDL in subjects with T2DM. rHDL showed similar function when compared with native HDL, while the G-HDL failed to activate the signaling pathway. rHDL showed the most effect in activation of the pathway at each tested time point (Figure 5A, B and C, P < 0.01), suggesting a selective effect of S1P in mediating these effects. The phosphorylation was triggered by the three types of HDL starting at 5 minute and reached the peak at 15 minutes, and returned to baseline at 60 minutes. The effect of rHDL in activation of the phosphorylation of ERK and MAPK was reduced by the VPC23019 (Figure 5D, E and F) suggesting that this effect was mediated by the S1P receptors.

### Reconstituted HDL enhances the transcription factor CREB binding on the cis-inducible element (CIE) DNA

**Figure 6:**
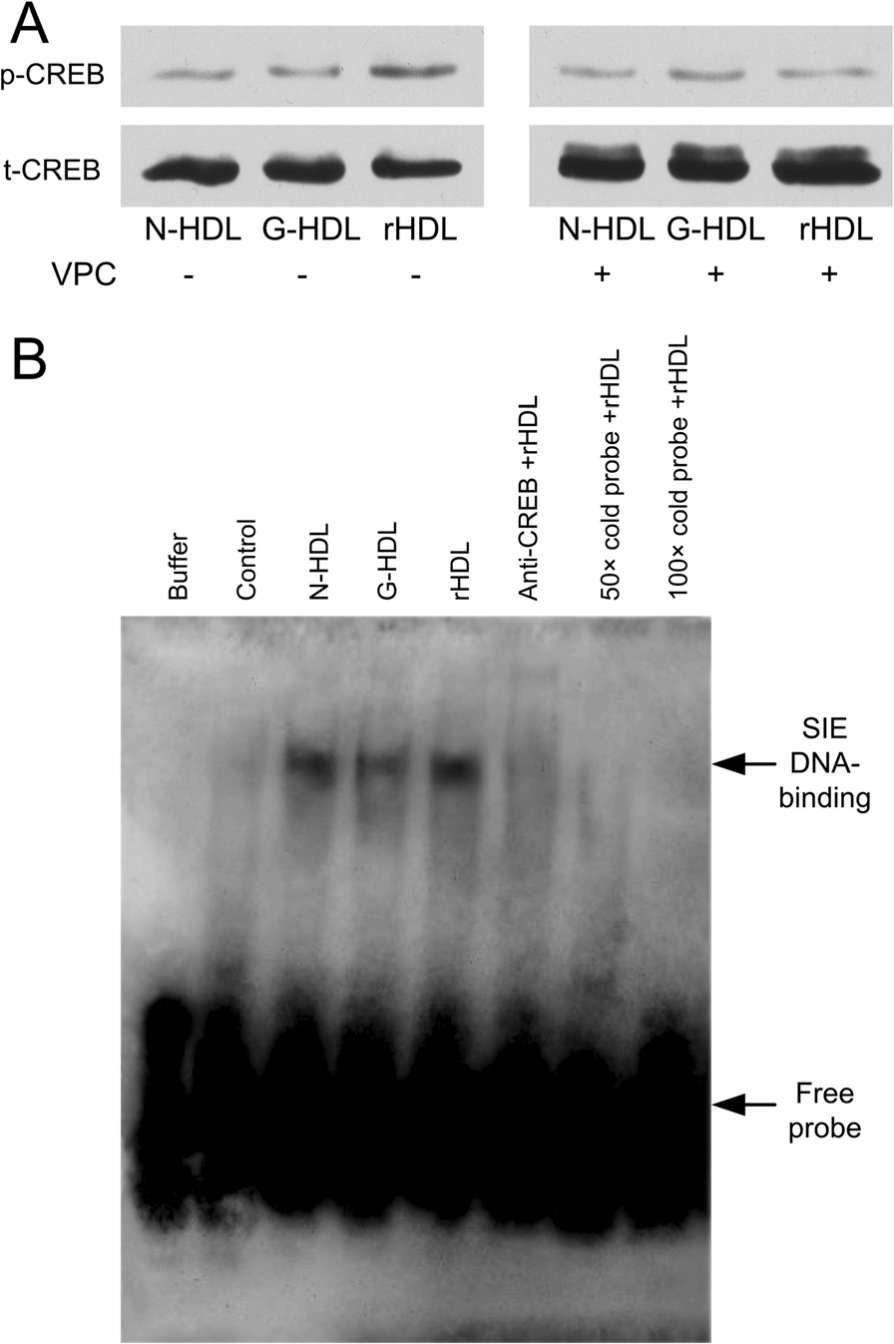
**The phosphorylation and activation of CERB was induced by N-HDL, G-HDL, rHDL (G-HDL containing S1P). A-C**: HUVECs were incubated with 30 μg/ml of N-HDL, G-HDL, rHDL at 15 minute and the activation of CREB was analyzed by Western blotting, HUVECs were pre-incubated with VPC 23019 (15 nmol/ml) for 20 minute and further treated 30 μg/ml of N-HDL, G-HDL, rHDL at 15 minute and the activation of CREB was analyzed by Western blotting **(A)** and the activation of transcription factor CERB at 15 minute was determined by EMSA **(B)**. Each experiment was repeated three times.

Since ERK1/2 and MAPK were the modulators of the CREB protein, we tested whether the three kinds of HDL enhanced the nuclear transcription factor CREB activity or not. rHDL and native HDL markedly increased CREB phosphorylation, whereas G-HDL had a considerably weaker effect and antagonist of S1PR1 and S1PR3 reduced effects of rHDL (Figure 6A). As shown in Figure 6B, reconstituted HDL enhanced more transcription factor binding on the CIE DNA than the other two forms of HDL. The binding of CREB to the CIE sequence disappeared with 50-fold and 100-fold excess of unlabeled CIE DNA (cold probe), confirming the specificity of CIE DNA binding. CREB binding with CIE as CREB/CIE DNA complex got abolished when anti-CREB antibody was added in the system. These results demonstrate that extra S1P loading on glycated HDL induced more transcription factor CREB binding on its CIE DNA complex.

## Discussion

Diabetes mellitus is associated with HDL dysfunction which accelerated atherosclerosis, in which HDL particle undergoes diverse structural modifications resulting in significant changes in its composition and function [6]. In this study, we investigated one of the most common modifications, glycation and a well-known bioactive lipid molecule, S1P.

Previous studies have shown that non-enzymic glycation was a common modification that appeared in T2DM and this modification impaired HDL’s anti-inflammatory and anti-oxidant roles [33]. In this study, we explored the mechanism involved in the effect of S1P in repairing the function of glycated HDL, like inducing COX-2 expression and PGI-2 release. We have previously shown that the level of diabetic HDL-associated S1P is increased compared with native HDL [25]. As the following work of that research, we found out that in the T2DM group, the level of S1P harbored on HDL in T2DM patients decreased with the elevated level of HbA1c. So we assumed that higher level of S1P on HDL could serve as protective effects to the vascular endothelial, but with the progress of T2DM, the level of S1P was decreased and the function of it was also diminished. At the same time, we tried to figure out the potential mechanism involved in the increased level of S1P associated with diabetic HDL [25]. The explanation of the increased level of diabetic HDL-associated S1P was related to its levels of expression of key elimination enzymes. S1P is generated by SphK1/2 from sphingosine and eliminated by dephosphorylation and lyase reactions [34]. The dephosphorylation of S1P is catalyzed by the actions of S1P-specific phosphatases, as well as by enzymes of the nonspecific lipid phosphate phosphatase family (LPPs). Each of these enzymes regenerates sphingosine which can be rapidly re-phosphorylated, thereby restoring S1P levels. In contrast to the phosphatases, SPL catalyzes an irreversible cleavage reaction that provides global control over circulating S1P levels and tissue S1P pools [14,15]. As the final enzyme in the sphingolipid degradation pathway, SPL controls the only exit point for sphingolipid intermediates and their flow into phospholipid metabolism [34]. Thus, in addition to regulating S1P levels, SPL is the gatekeeper of a critical node of lipid metabolic flow. The SPL plays the key role in regulation of the S1P level in plasma and recruitment back to sphingosine and thereby influencing S1P-dependent biological and pathological processes [24]. In our study, we found SPL activity is decreased which perhaps leads to the increased level of HDL-associated S1P increased in T2DM [25]. Previous studies were shown that S1P was preferentially associating with HDL3 [35]. In our work, we confirmed that the increasing the level of HDL3-associated S1P restores its function similar to what is observed with HDL from diabetic subjects. This suggests that perhaps in T2DM the increased levels of SIP may be a compensatory protective mechanism.

The role of S1P in atherosclerosis is complex, as it has both pro- and anti-atherosclerotic properties as shown in previous studies [36,37]. The endothelial system sets up the first barrier for the blood vessels and HDL with S1P plays the important role in this protective procedure. S1P receptors are G-coupled receptors located on endothelial membrane where S1PR1 and S1PR3 are mainly located. So the increased level of S1P occurring in diabetic HDL3 may have vascular protective effects. The accumulation of the lipids on HDL, especially S1P, compensates the functional loss and may exert the role of protection by regulation of COX-2 expression and activation of ERK/MAPK-CREB pathway.

In addition to increased risk of stroke, myocardial infarction, and peripheral vascular disease, diabetics suffer from a particularly aggressive form of atherosclerosis with greater in-hospital mortality following myocardial infarction, a higher incidence of heart failure and stroke [6]. The prevention of complications needs more focus.

## Conclusions

The current study demonstrates that the increasing level of S1P occurring in diabetic HDL3 have vascular protective effects. The accumulation of the lipids on HDL, especially S1P, compensates the functional loss and may exert the role of protection by regulation of COX-2 expression and activation of ERK/MAPK-CREB pathway. Such increased S1P concentration possibly results from the down-regulation of its degeneration enzymes (SPL and SGPP).

Our work sheds more light on the intrinsic mechanism behind the protective effects of HDL in diabetic disease thus opening doors for new therpeutic targets. Since currently available drug use in clinical practice has not provided us with appreciable results, new targets and insights are needed as soon as new disease mechanisms are recognized. The S1P pathway activation provides a potential site for future drug design.

## Abbreviations

T2DM: Type2 diabetes mellitus; HDL: High-density lipoprotein; rHDL: Reconstituted HDL; S1P: Sphingosine-1-phosphate; COX-2: Cyclooxygenase-2; PGI-2: Prostacyclin I-2; HUVECs: Human umbilical vein endothelial cells; S1PR1: S1P receptor 1; S1PR3: S1P receptor 3; apoHDL&PL: HDL containing the protein components and the phospholipids; SGPP: S1P-specific phosphatases; SPL: Sphingosine-1-phosphate lyase.

## Competing interests

The authors declare they have no competing interests.

## Authors’ contribution

As to the contribution of each author, XT, LZ and YH designed the study, analyzed and interpreted the results, and drafted the manuscript; PLv, DL, CN, YW, LJ, JL, ZF made substantial contributions to performing the experimental protocol; AVM and SP were involved in revising the manuscript critically for important intellectual content. All authors participated in the discussion and interpretation of the results and in the final approval of the manuscript submitted.

## Supplementary Material

Additional file 1: Figure S1 Detection of MDA level and PON1 activity in N-HDL and G-HDL by spectrophotometry. The differences of MDA level and PON1 activity in the two groups have no statistical significance. **Figure S2.** The level of S1P reconstituted on HDL (30 mg/ml) was detected by the method of UPLC-MS (A). The levels of S1P we reconstituted were nearly equal to the S1P we detected on reconstituted HDL. **Figure S3.** In the T2DM group, levels of S1P harbored on HDL (Y axis) were decreased when the levels of HbA1c (X axis) increased. The correlation coefficient R was 0.7574, P < 0.001. Click here for file

## References

[B1] Strojek K Features of macrovascular complications in type 2 diabetic patients Acta Diabetol 2003 40 Suppl 2 S334 S337 14704864 10.1007/s00592-003-0115-x

[B2] Plutzky J Viberti G Haffner S Atherosclerosis in type 2 diabetes mellitus and insulin resistance: mechanistic links and therapeutic targets J Diabetes Complications 2002 16 6 401 415 10.1016/S1056-8727(02)00202-7 12477625

[B3] Neeli H Gadi R Rader DJ Managing diabetic dyslipidemia: beyond statin therapy Curr Diab Rep 2009 9 1 11 17 10.1007/s11892-009-0004-y 19192419

[B4] Drew BG Rye KA Duffy SJ Barter P Kingwell BA The emerging role of HDL in glucose metabolism Nat Rev Endocrinol 2012 8 4 237 245 10.1038/nrendo.2011.235 22271188

[B5] Barter P Gotto AM LaRosa JC Maroni J Szarek M Grundy SM Kastelein JJ Bittner V Fruchart JC HDL cholesterol, very low levels of LDL cholesterol, and cardiovascular events N Engl J Med 2007 357 13 1301 1310 10.1056/NEJMoa064278 17898099

[B6] Mooradian AD Dyslipidemia in type 2 diabetes mellitus Nat Clin Pract Endocrinol Metab 2009 5 3 150 159 10.1038/ncpendmet1066 19229235

[B7] Cipollone F Cicolini G Bucci M Cyclooxygenase and prostaglandin synthases in atherosclerosis: recent insights and future perspectives Pharmacol Ther 2008 118 2 161 180 10.1016/j.pharmthera.2008.01.002 18420277

[B8] Karaoglu A Tunc T Aydemir G Onguru O Uysal B Kul M Aydinoz S Oztas E Sarici U Role of cyclooxygenase 2 and endothelial nitric oxide synthetase in preclinical atherosclerosis Fetal Pediatr Pathol 2012 31 6 432 438 10.3109/15513815.2012.659408 22443285

[B9] Damirin A Tomura H Komachi M Tobo M Sato K Mogi C Nochi H Tamoto K Okajima F Sphingosine 1-phosphate receptors mediate the lipid-induced cAMP accumulation through cyclooxygenase-2/prostaglandin I2 pathway in human coronary artery smooth muscle cells Mol Pharmacol 2005 67 4 1177 1185 10.1124/mol.104.004317 15625281

[B10] Rodriguez C Gonzalez-Diez M Badimon L Martinez-Gonzalez J Sphingosine-1-phosphate: a bioactive lipid that confers high-density lipoprotein with vasculoprotection mediated by nitric oxide and prostacyclin Thromb Haemost 2009 101 4 665 673 19350109

[B11] Argraves KM Argraves WS HDL serves as a S1P signaling platform mediating a multitude of cardiovascular effects J Lipid Res 2007 48 11 2325 2333 10.1194/jlr.R700011-JLR200 17698855

[B12] Bourquin F Capitani G Grutter MG PLP-dependent enzymes as entry and exit gates of sphingolipid metabolism Protein Sci 2011 20 9 1492 1508 10.1002/pro.679 21710479 PMC3190145

[B13] Kono M Mi Y Liu Y Sasaki T Allende ML Wu YP Yamashita T Proia RL The sphingosine-1-phosphate receptors S1P1, S1P2, and S1P3 function coordinately during embryonic angiogenesis J Biol Chem 2004 279 28 29367 29373 10.1074/jbc.M403937200 15138255

[B14] Aguilar A Saba JD Truth and consequences of sphingosine-1-phosphate lyase Adv Biol Regul 2012 52 1 17 30 10.1016/j.advenzreg.2011.09.015 21946005 PMC3560305

[B15] Serra M Saba JD Sphingosine 1-phosphate lyase, a key regulator of sphingosine 1-phosphate signaling and function Adv Enzyme Regul 2010 50 1 349 362 10.1016/j.advenzreg.2009.10.024 19914275 PMC2862839

[B16] Matsuki K Tamasawa N Yamashita M Tanabe J Murakami H Matsui J Imaizumi T Satoh K Suda T Metformin restores impaired HDL-mediated cholesterol efflux due to glycation Atherosclerosis 2009 206 2 434 438 10.1016/j.atherosclerosis.2009.03.003 19376519

[B17] Nobecourt E Zeng J Davies MJ Brown BE Yadav S Barter PJ Rye KA Effects of cross-link breakers, glycation inhibitors and insulin sensitisers on HDL function and the non-enzymatic glycation of apolipoprotein A-I Diabetologia 2008 51 6 1008 1017 10.1007/s00125-008-0986-z 18437350

[B18] Liu D Ji L Zhang D Tong X Pan B Liu P Zhang Y Huang Y Su J Willard B Zheng L Nonenzymatic glycation of high-density lipoprotein impairs its anti-inflammatory effects in innate immunity Diabetes Metab Res Rev 2012 28 2 186 195 10.1002/dmrr.1297 21928330

[B19] Zheng L Nukuna B Brennan ML Sun M Goormastic M Settle M Schmitt D Fu X Thomson L Fox PL Ischiropoulos H Smith JD Kinter M Hazen SL Apolipoprotein A-I is a selective target for myeloperoxidase-catalyzed oxidation and functional impairment in subjects with cardiovascular disease J Clin Invest 2004 114 4 529 541 10.1172/JCI200421109 15314690 PMC503769

[B20] Zheng L Settle M Brubaker G Schmitt D Hazen SL Smith JD Kinter M Localization of nitration and chlorination sites on apolipoprotein A-I catalyzed by myeloperoxidase in human atheroma and associated oxidative impairment in ABCA1-dependent cholesterol efflux from macrophages J Biol Chem 2005 280 1 38 47 15498770 10.1074/jbc.M407019200

[B21] Pan B Ma Y Ren H He Y Wang Y Lv X Liu D Ji L Yu B Wang Y Chen YE Pennathur S Smith JD Liu G Zheng L Diabetic HDL is dysfunctional in stimulating endothelial cell migration and proliferation due to down regulation of SR-BI expression PLoS One 2012 7 11 e48530 10.1371/journal.pone.0048530 23133640 PMC3487724

[B22] Baranowski M Blachnio-Zabielska A Hirnle T Harasiuk D Matlak K Knapp M Zabielski P Gorski J Myocardium of type 2 diabetic and obese patients is characterized by alterations in sphingolipid metabolic enzymes but not by accumulation of ceramide J Lipid Res 2010 51 1 74 80 10.1194/jlr.M900002-JLR200 19617631 PMC2789788

[B23] Saba JD Hla T Point-counterpoint of sphingosine 1-phosphate metabolism Circ Res 2004 94 6 724 734 10.1161/01.RES.0000122383.60368.24 15059942

[B24] Hannun YA Obeid LM Principles of bioactive lipid signalling: lessons from sphingolipids Nat Rev Mol Cell Biol 2008 9 2 139 150 10.1038/nrm2329 18216770

[B25] Tong X Peng H Liu D Ji L Niu C Ren J Pan B Hu J Zheng L Huang Y High-density lipoprotein of patients with Type 2 Diabetes Mellitus upregulates cyclooxgenase-2 expression and prostacyclin I-2 release in endothelial cells: relationship with HDL-associated sphingosine-1-phosphate Cardiovasc Diabetol 2013 12 1 27 10.1186/1475-2840-12-27 23360427 PMC3599898

[B26] Jaffe EA Nachman RL Becker CG Minick CR Culture of human endothelial cells derived from umbilical veins. Identification by morphologic and immunologic criteria J Clin Invest 1973 52 11 2745 2756 10.1172/JCI107470 4355998 PMC302542

[B27] Chung BH Wilkinson T Geer JC Segrest JP Preparative and quantitative isolation of plasma lipoproteins: rapid, single discontinuous density gradient ultracentrifugation in a vertical rotor J Lipid Res 1980 21 3 284 291 7381323

[B28] Cham BE Knowles BR **A solvent system for delipidation of plasma or serum without protein precipitation** J Lipid Res 1976 17 2 176 181 818332

[B29] Lee MH Hammad SM Semler AJ Luttrell LM Lopes-Virella MF Klein RL HDL3, but not HDL2, stimulates plasminogen activator inhibitor-1 release from adipocytes: the role of sphingosine-1-phosphate J Lipid Res 2010 51 9 2619 2628 10.1194/jlr.M003988 20522601 PMC2918445

[B30] Liu D Ji L Tong X Pan B Han JY Huang Y Chen YE Pennathur S Zhang Y Zheng L Human apolipoprotein A-I induces cyclooxygenase-2 expression and prostaglandin I-2 release in endothelial cells through ATP-binding cassette transporter A1 Am J Physiol Cell Physiol 2011 301 3 739 748 10.1152/ajpcell.00055.2011 21734188

[B31] Burnette WN "Western blotting": electrophoretic transfer of proteins from sodium dodecyl sulfate–polyacrylamide gels to unmodified nitrocellulose and radiographic detection with antibody and radioiodinated protein A Anal Biochem 1981 112 2 195 203 10.1016/0003-2697(81)90281-5 6266278

[B32] Read JT Cheng H Hendy SC Nelson CC Rennie PS Receptor-DNA interactions: EMSA and footprinting Methods Mol Biol 2009 505 97 122 10.1007/978-1-60327-575-0_6 19117141

[B33] Verges B New insight into the pathophysiology of lipid abnormalities in type 2 diabetes Diabetes Metab 2005 31 5 429 439 10.1016/S1262-3636(07)70213-6 16357786

[B34] Spiegel S Milstien S The outs and the ins of sphingosine-1-phosphate in immunity Nat Rev Immunol 2011 11 6 403 415 10.1038/nri2974 21546914 PMC3368251

[B35] Kontush A Therond P Zerrad A Couturier M Negre-Salvayre A de Souza JA Chantepie S Chapman MJ Preferential sphingosine-1-phosphate enrichment and sphingomyelin depletion are key features of small dense HDL3 particles: relevance to antiapoptotic and antioxidative activities Arterioscler Thromb Vasc Biol 2007 27 8 1843 1849 10.1161/ATVBAHA.107.145672 17569880

[B36] Keul P Lucke S von Wnuck LK Bode C Graler M Heusch G Levkau B Sphingosine-1-phosphate receptor 3 promotes recruitment of monocyte/macrophages in inflammation and atherosclerosis Circ Res 2011 108 3 314 323 10.1161/CIRCRESAHA.110.235028 21164103

[B37] Whetzel AM Bolick DT Srinivasan S Macdonald TL Morris MA Ley K Hedrick CC Sphingosine-1 phosphate prevents monocyte/endothelial interactions in type 1 diabetic NOD mice through activation of the S1P1 receptor Circ Res 2006 99 7 731 739 10.1161/01.RES.0000244088.33375.52 16960101

