# Supplementary material for: The compensatory enrichment of sphingosine -1- phosphate harbored on glycated high-density lipoprotein restores endothelial protective function in type 2 diabetes mellitus
Source: Cardiovasc Diabetol. 2014 Apr 21;13:82. doi: 10.1186/1475-2840-13-82 (PMC4021293; doi:10.1186/1475-2840-13-82)
Supplement: Additional file 1: Figure S1 — Detection of MDA level and PON1 activity in N-HDL and G-HDL by spectrophotometry. The differences of MDA level and PON1 activity in the two groups have no statistical significance. Figure S2. The level of S1P reconstituted on HDL (30 mg/ml) was detected by the method of UPLC-MS (A). The levels of S1P we reconstituted were nearly equal to the S1P we detected on reconstituted HDL. Figure S3. In the T2DM group, levels of S1P harbored on HDL (Y axis) were decreased when the levels of HbA1c (X axis) increased. The correlation coefficient R was 0.7574, P < 0.001. [file 1475-2840-13-82-S1.docx]

**Supplement Method**

**Subjects**

30 patients with T2DM were newly recruited following the inclusive criteria. HDL of these patients were isolated and S1P was extracted as the methods we mentioned above.

**Detection of MDA in HDL by spectrophotometry.**

MDA in HDL was detected by a commercially available lipid peroxidation assay kit (ALDetect, Enzo Life Sciences) involving an acid hydrolysis step in the presence of butylatedhydroxytoluene (BHT). Hydrolysis of Schiff base MDA adducts in HDL was carried out after addition of hydrochloric acid at pH 1–2 and heating at 60°C for 80 minutes. Other steps were followed the structure of the commercial kit.

**Measurement of PON1 activity of HDL.**

Activities of HDL-associated PON1 were measured by spectrophotometry using the substrates paraoxon and phenylacetate, respectively.

**Supplement Figure 1**

Detection of MDA level and PON1 activity in N-HDL and G-HDL by spectrophotometry. The differences of MDA level and PON1 activity in the two groups have no statistical significant.





**Supplement Figure 2**

The level of S1P reconstituted on HDL (30mg/ml) was detected by the method of UPLC-MS (A). The levels of S1P we reconstituted were nearly equals to the S1P we detected on reconstituted HDL.





**Supplement Figure 3**

In the T2DM group, levels of S1P harbored on HDL(Y axis) were decreased accompanied with the levels of HbA1c (X axis) increased. The correlation coefficient R was 0.7574, P< 0.001.
